# Recent Workmen's Compensation Cases

**Published:** 1910-01-15

**Authors:** 


### The Hospital

A Journal of

The Medical Sciences and Hospital Administration.

New Series. No. 152, Vol. VI. [No. 1224, Vol. XLVII.] SATURDAY, JANUARY 15, 1910.

### Recent Workmen's Compensation Cases.

Close attention to the decisions of cases relating to Workmen's Compensation is almost as necessary for the doctor as it is for the lawyer. Hundreds of cases have been decided wholly by medical evidence, and if the practitioner is familiar with the more important cases dealing with the subject, it is obvious that he can so much the better advise his client.

Recently the County Court judges have had to consider many difficult points. In one case it appeared that a stoker met with his death in the stokehold of a ship. The medical evidence for the applicant was that death was caused by cerebral hæmorrhage as the result of excessive heat in the stokehold and the copious amount of water drunk by the deceased. On the other hand, medical witnesses on behalf of the employers said that cerebral hæmorrhage could not have been the result of heat from the furnaces. Heatstroke was not uncommon among stokers, but that was not the cause of death. In the result the Court of Appeal decided in favour of the widow. The numerous cases heard in various county courts throughout the country have shed a light upon the meaning of the term "accident" as used in the Compensation Act. One thing is clear: accident must be proved; it cannot be assumed. So where a man who was employed on board a ship in dock was found drowned in the water alongside, and there was no evidence to show why he fell into the water, the shipowners were held not liable. In another case a man was poisoned by carbolic acid. It was proved that this substance was used as a disinfectant where he worked, and that a bottle containing traces of it was found near where he had been lying. It was in a bottle out of which the men at the works sometimes took their drinks. A claim for compensation was dismissed on the ground that there was nothing to show why the man took the acid.

Amongst miners the disease known as "nystagmus" has frequently been made the ground of a claim for compensation. Being a recognised "industrial disease," claims made in respect of it are subject to the rule that if the workman has been employed in more than one pit, the compensation payable must be divided up in proportion to the time worked in each. This was what actually happened in one case at Pontypridd. Medical evidence showed that the applicant for compensation had been suffering from the complaint in question for twelve months. As he had been at four different pits in that time, the judge ordered that the lump sum awarded should be divided up in proportion to the time served at each pit.

In a case heard at Bradford an application was made on behalf of a man who was alleged to have become insane owing to lead poisoning, which is a recognised industrial disease. Several medical witnesses were called, who said that in their opinion the insanity was due to lead poisoning, whilst several others took the opposite view. The learned judge, after considering what opportunity each witness had had for forming his opinion, and their experience in mental cases, came to the conclusion that the lead poisoning did contribute to the insanity. He therefore gave judgment for the applicant. In another case a workman, who was employed for many years as a painter, died from granular kidney. This disease is a sequela of lead poisoning, but it is also a sequela of gout, alcoholism, and other complaints. In proceedings for the assessment of compensation under the Act it was not proved that lead poisoning was the cause of the granular kidney, but the employers did not prove that it was not the cause.

While the Court has no power to order a workman to undergo an operation in order to increase his working capacity, it can do so indirectly by making a suspensory award. Thus it may order that unless he takes reasonable steps to improve his working capacity, he shall only receive a nominal sum per week. The principle upon which the Court will act in these cases has been clearly laid down in the Court of Appeal, where the Master of the Rolls said: "The test is whether the man in refusing to undergo the operation acted unreasonably. I decline to admit that a man can be said to have acted unreasonably when he followed the advice of a competent doctor. . . . After the evidence of the man's doctor no amount of medical evidence is relevant to this question." The principle of the above decision has been adopted in several recent cases. Thus, in a case heard at Doncaster on June 23, it appeared that a man aged 54 had his foot run over by a cart. He was in hospital for four months and underwent three operations, receiving weekly compensation down to January 15, 1909. On that date the employers refused to continue payment unless he would undergo a fourth operation, which they were advised would give him a useful limb. Two doctors said the operation was attended with risk. Dr. Pye-Smith (of Sheffield) said the operation could be successfully performed, but he agreed "that the question of the advisability of an operation was one on which a competent man might hold a contrary opinion." In these circumstances the judge declined to compel the workman to undergo a further operation.

Apart from operation, an injured workman must submit himself to proper medical treatment. Thus in one case the Court of Appeal suspended an order for compensation where a man would not wear a truss. The evidence was that if he did wear a truss he could earn his wages. In a case heard at Sheffield, it appeared that a man had to have his little finger amputated because of an accident. After the wound had healed the surgeon advised a course of massage, in order to remove a certain stiffness which had set in owing to the non-use of the hand. According to the evidence, the man had undergone the massage, but had not, in the opinion of the doctor, made a proper effort to use his hand. "It requires," said the doctor, in addition to the massage, "a certain exercise of will power." The judge, however, held that as the man had been treated by massage as advised, the fact of his not having exercised sufficient will power to get well did not disentitle him to an award. In another case, heard at Walsall, it was alleged that the workman had prevented his own recovery by not submitting to an operation for the breaking-down of fibrous adhesions which prevented movement of the ankle. A report to this effect had been supplied by a medical referee. The judge held that the man had prevented his own recovery by not submitting to a reasonable operation in the earlier stages of his treatment, and determined the award. In another case (heard at Doncaster) where the applicant had had his arm scalded, the judge suggested in the course of the case that he should undergo a course of treatment in hospital, and allowed the case to stand over in order to see how far this treatment was likely to be successful.

